# Bringing Access to the Full Spectrum of Cancer Research: A Call for Papers

**DOI:** 10.1371/journal.pmed.1001817

**Published:** 2015-04-17

**Authors:** The PLOS Medicine Editors

## Abstract

The *PLOS Medicine* editors issue a call for papers for translational and clinical cancer research papers with strong potential to advance patient care, public policy, or clinical research agendas.

Cancer is a keyword in innumerable stories of both loss and hope. In 2012, 14.1 million people were diagnosed with cancer worldwide, with an estimated 8.2 million deaths annually [1]. The disease burden is daunting, yet long-term investment of resources in research is now yielding cause for optimism. For example, the United States Food and Drug Administration (FDA) has approved over 30 cancer drugs within the past three years [2], many of which were designed in response to insights gained from genomic investigations and developed to specifically target a gene or protein that is required for tumor growth, spread, or survival.

Understanding the clonal evolution of a cancer and the mutations that accrue over time could result in even more precise therapies. Recent findings from the cancer genomics field have also revealed the extent to which cancers are not just heterogeneous among people but also within an individual [3,4]. Propelled by improved technologies such as single-cell sequencing, longitudinal studies on an individual can trace a cancer’s development over time and through metastases, providing guidance for potentially individualized therapies.

As an open-access journal committed to publishing outstanding research and commentaries on the major challenges to human health worldwide, *PLOS Medicine* endeavors to provide a prominent venue for publishing global cancer research. With growing and aging populations, low- and middle-income countries (LMICs) are disproportionately affected by the increasing numbers of cancers worldwide, with more than 60% of the world’s total cancer cases and 70% of the deaths occurring in Africa, Asia, and Central and South America [1]. In lower-resource settings, the situation is made worse by the lack of early detection and access to treatment. Recent Policy Forum articles in *PLOS Medicine* have outlined key components to the establishment of national childhood cancer strategies in LMICs [5] and strategies to improve cancer care for cervical cancer in LMICs, including scale-up of human papilloma virus (HPV) vaccination and integration of care and prevention services with HIV and maternal services [6]. Given that disparities also exist in access to cancer care within high-income countries (e.g., for lung cancer treatment [7]), *PLOS Medicine* welcomes submissions of research that provides solutions to access to care within both low- and high-resource settings.

Reducing the burden of cancer means addressing the full spectrum of contributing conditions. Accordingly, in recent months, *PLOS Medicine* has published research on topics ranging from identifying and improving environmental and lifestyle-related exposures [8,9,10] to optimizing the benefits and reducing the harms of screening [11,12,13,14], as well as translating basic science towards state-of-the art treatments [3,4,15,16] and ensuring that such interventions are available to all who need them [7,17].

Within this spectrum, the editorial team believes that translational genomics represents a “state of the art” wavelength, able to illuminate both basic pathophysiology and therapeutic decisions and options. The editorial team is actively engaging with the cancer genomics community, both through our academic editors, such as Andrew Beck (Dana-Farber/ Harvard Cancer Center) who discussed the importance of open access to cancer genomics data in a recent editorial [18], and also via our attendance at key conferences in 2015. Two consortia that provide broad public access to cancer sequence data are The Cancer Genome Atlas (TCGA, http://cancergenome.nih.gov/) and International Cancer Genome Consortium (ICGC, https://icgc.org/). We are very pleased to feature an accompanying interview blog [19] with Francis Ouellette, Associate Director of Informatics and Biocomputing at the Ontario Institute for Cancer Research, who discusses the remit of the ICGC and TCGA projects, how these projects have generated a tidal wave of data that has reshaped how people consider analyzing such datasets, and his hopes for how these findings will translate into clinical applications.

To coincide with the meeting of the American Association for Cancer Research (AACR), to be held in Philadelphia from April 18 to 22, 2015, *PLOS Medicine* is launching a Cancer Research Collection [20], an open-access collection of recently published articles representing the full spectrum of clinically relevant cancer research and commentary, from translational to clinical to epidemiological. To expand the collection, and to support the mission we share with the AACR to conquer cancer through research and education, we are issuing a call to the clinical genomics and cancer research community for papers that provide novel insights into cancer heterogeneity, progression, and translational and clinical medicine, with strong potential to advance patient care, public policy, or clinical research agendas. Papers submitted in response to the call for papers will be included in the collection if accepted for publication. Please submit a presubmission inquiry to the editorial team at http://www.editorialmanager.com/pmedicine/default.aspx.

## Abbreviations

AACR: American Association for Cancer Research
FDA: Food and Drug Administration
HPV: human papilloma virus
ICGC: International Cancer Genome Consortium
LMICs: low- and middle-income countries
TCGA: The Cancer Genome Atlas

## References

[1] Stewart BW, Wild CP, editors (2014) World Cancer Report 2014. http://www.iarc.fr/en/publications/books/wcr/index.php. International Agency for Research on Cancer.

[2] US FDA (2015) Hematology/Oncology (Cancer) Approvals & Safety Notifications. http://www.fda.gov/Drugs/InformationOnDrugs/ApprovedDrugs/ucm279174.htm. Accessed 17 March 2015.

[3] Schwarz RF, Ng CKY, Cooke SL, Newman S, Temple J, et al (2015) Spatial and Temporal Heterogeneity in High-Grade Serous Ovarian Cancer: A Phylogenetic Analysis. PLoS Med 12(2): e1001789 10.1371/journal.pmed.1001789 25710373 PMC4339382

[4] Mroz EA, Tward AM, Hammon RJ, Ren Y, Rocco JW (2015) Intra-tumor Genetic Heterogeneity and Mortality in Head and Neck Cancer: Analysis of Data from The Cancer Genome Atlas. PLoS Med 12(2): e1001786 10.1371/journal.pmed.1001786 25668320 PMC4323109

[5] Gupta S, Rivera-Luna R, Ribeiro RC, Howard SC (2014) Pediatric Oncology as the Next Global Child Health Priority: The Need for National Childhood Cancer Strategies in Low- and Middle-Income Countries PLoS Med 11: e1001656 10.1371/journal.pmed.1001656 24936984 PMC4061014

[6] Singhrao R, Huchko M, Yamey G (2013) Reproductive and Maternal Health in the Post-2015 Era: Cervical Cancer Must Be a Priority. PLOS Med 10: e1001499 10.1371/journal.pmed.1001499 23966840 PMC3742438

[7] Forrest LF, Adams J, Wareham H, Rubin G, White M (2013) Socioeconomic Inequalities in Lung Cancer Treatment: Systematic Review and Meta-Analysis. PLoS Med 10(2): e1001376 10.1371/journal.pmed.1001376 23393428 PMC3564770

[8] Kitahara CM, Flint AJ, Berrington de Gonzalez A, Bernstein L, Brotzman M, et al (2014) Association between Class III Obesity (BMI of 40–59 kg/m2) and Mortality: A Pooled Analysis of 20 Prospective Studies. PLoS Med 11(7): e1001673 10.1371/journal.pmed.1001673 25003901 PMC4087039

[9] Ulucanlar S, Fooks GJ, Hatchard JL, Gilmore AB (2014) Representation and Misrepresentation of Scientific Evidence in Contemporary Tobacco Regulation: A Review of Tobacco Industry Submissions to the UK Government Consultation on Standardised Packaging. PLoS Med 11(3): e1001629 10.1371/journal.pmed.1001629 24667150 PMC3965396

[10] Zheng W, McLerran DF, Rolland BA, Fu Z, Boffetta P, et al (2014) Burden of Total and Cause-Specific Mortality Related to Tobacco Smoking among Adults Aged ≥45 Years in Asia: A Pooled Analysis of 21 Cohorts. PLoS Med 11(4): e1001631 10.1371/journal.pmed.1001631 24756146 PMC3995657

[11] Singal AG, Pillai A, Tiro J (2014) Early Detection, Curative Treatment, and Survival Rates for Hepatocellular Carcinoma Surveillance in Patients with Cirrhosis: A Meta-analysis. PLoS Med 11(4): e1001624 10.1371/journal.pmed.1001624 24691105 PMC3972088

[12] Castañón A, Landy R, Cuzick J, Sasieni P (2014) Cervical Screening at Age 50–64 Years and the Risk of Cervical Cancer at Age 65 Years and Older: Population-Based Case Control Study. PLoS Med 11(1): e1001585 10.1371/journal.pmed.1001585 24453946 PMC3891624

[13] Tammemägi MC, Church TR, Hocking WG, Silvestri GA, Kvale PA, et al (2014) Evaluation of the Lung Cancer Risks at Which to Screen Ever- and Never-Smokers: Screening Rules Applied to the PLCO and NLST Cohorts. PLoS Med 11(12): e1001764 10.1371/journal.pmed.1001764 25460915 PMC4251899

[14] Ross-Innes CS, Debiram-Beecham I, O'Donovan M, Walker E, Varghese S, et al (2015) Evaluation of a Minimally Invasive Cell Sampling Device Coupled with Assessment of Trefoil Factor 3 Expression for Diagnosing Barrett's Esophagus: A Multi-Center Case–Control Study. PLoS Med 12(1): e1001780 10.1371/journal.pmed.1001780 25634542 PMC4310596

[15] Kim JH, Sohn BH, Lee H-S, Kim S-B, Yoo JE, et al (2014) Genomic Predictors for Recurrence Patterns of Hepatocellular Carcinoma: Model Derivation and Validation. PLoS Med 11(12): e1001770 10.1371/journal.pmed.1001770 25536056 PMC4275163

[16] Martinez-Torres A-C, Quiney C, Attout T, Boullet H, Herbi L, et al (2015) CD47 Agonist Peptides Induce Programmed Cell Death in Refractory Chronic Lymphocytic Leukemia B Cells via PLCγ1 Activation: Evidence from Mice and Humans. PLoS Med 12(3): e1001796 10.1371/journal.pmed.1001796 25734483 PMC4348493

[17] Eng A, McCormack V, dos-Santos-Silva I (2014) Receptor-Defined Subtypes of Breast Cancer in Indigenous Populations in Africa: A Systematic Review and Meta-Analysis. PLoS Med 11(9): e1001720 10.1371/journal.pmed.1001720 25202974 PMC4159229

[18] Beck AH (2015) Open Access to Large Scale Datasets Is Needed to Translate Knowledge of Cancer Heterogeneity into Better Patient Outcomes. PLoS Med 12(2): e1001794 10.1371/journal.pmed.1001794 25710538 PMC4339838

[19] *PLOS Medicine* Editors (2015 4 17) Cancer genomics: data, data and more data. In: Speaking of Medicine Blog. Available: http://blogs.plos.org/speakingofmedicine/2015/04/17/interview-francis-ouellette/

[20] *PLOS Medicine* Editors (2015) Cancer Research Collection homepage. Available: www.ploscollections.org/plosmedicinecancerreasearch

